# MYB96 shapes the circadian gating of ABA signaling in *Arabidopsis*

**DOI:** 10.1038/srep17754

**Published:** 2016-01-04

**Authors:** Hong Gil Lee, Paloma Mas, Pil Joon Seo

**Affiliations:** 1 Department of Bioactive Material Sciences and Research Center of Bioactive Materials, Chonbuk National University, Jeonju 561-756, Republic of Korea; 2 Plant Development and Signal Transduction, Center for Research in Agricultural Genomics (CRAG), Parc de Recerca UAB, Bellaterra (Cerdanyola del Vallés), Barcelona, Spain; 3 Consejo Superior de Investigaciones Científicas (CSIC), Barcelona, Spain; 4 Department of Chemistry and Research Institute of Physics and Chemistry, Chonbuk National University, Jeonju 561-756, Republic of Korea

## Abstract

Circadian clocks regulate the rhythms of biological activities with a period of approximately 24-hours and synchronize plant metabolism and physiology with the environmental cycles. The clock also gates responses to environmental stresses to maximize fitness advantages. Here we report that the MYB96 transcription factor is connected with the clock oscillator to shape the circadian gating of abscisic acid (ABA) responses. MYB96 directly binds to the *TIMING OF CAB EXPRESSION 1* (*TOC1*) promoter to positively regulate its expression. The use of *myb96* mutant plants shows that this regulation is essential for the gated induction of *TOC1* by ABA. In turn, *MYB96* induction by ABA is also altered in *toc1-3* mutant plants. The increased tolerance to drought of *MYB96* over-expressing plants is decreased in the *toc1-3* mutant background, suggesting that MYB96 and TOC1 intersect the circadian clock and ABA signaling. The MYB96-TOC1 function might be also regulated by the clock component CIRCADIAN CLOCK-ASSOCIATED 1 (CCA1), which binds to the *MYB96* promoter and alters its circadian expression. Thus, a complex circuitry of CCA1-MYB96-TOC1 regulatory interactions provides the mechanistic basis underlying the connection between circadian and stress signaling to optimize plant fitness to ambient stresses.

The circadian clock is a conserved biological time keeper mechanism that ensures endogenous rhythmic oscillation with a period of approximately 24 h. A sequential wave of negative transcriptional regulations defines the basic architecture of the circadian clock in *Arabidopsis*. Two single MYB repeat transcription factors, CIRCADIAN CLOCK-ASSOCIATED 1 (CCA1) and LATE ELONGATED HYPOCOTYL (LHY), and the TIMING OF CAB EXPRESSION 1/PSEUDO-RESPONSE REGULATOR 1 (TOC1/PRR1) comprise a central repressing regulatory module at the core of the clock^1^,^2^,^3^,^4^,^5^. Other regulatory loops are linked with this central module, including a morning loop, consisting of other members of the PRR family (PRR5, PRR7, and PRR9) and CCA1/LHY^6^,^7^, and the evening loop including a negative feedback regulation between PRR9 and the Evening Complex (EC) composed of EARLY FLOWERING3 (ELF3), ELF4, and LUX ARRYTHMO (LUX)^8^,^9^.

Precise clock function enables plants to synchronize endogenous physiological processes with the rhythmic environmental cycle, ensuring organism’s fitness and improved capability for environmental adaptation^10^,^11^,^12^. Notably, a large fraction of the plant transcriptome is estimated to be under the control of the circadian clock, and the clock-regulated genes are particularly over-represented in stress-responsive pathways^13^,^14^,^15^. Consistently, mis-expression of core clock components results in hypersensitivity to salt, osmotic, drought and heat stresses, indicating that the clock provides an efficient means for plants to anticipate the responses to environmental stresses^12^,^16^,^17^. In addition to the circadian regulation of stress responses, stress signaling also feedbacks into clock activity^18^. Many core clock genes contain *cis*-elements responsive to environmental stresses in their promoters^19^,^20^,^21^,^22^. The feedback regulation is important for timely gating the stress responses and also facilitates acute adaptive responses, balancing plant growth and energy-consuming stress tolerance.

The intimate relationship between ABA and the circadian signaling has been studied in detail. For instance, the bidirectional interaction between TOC1 and the putative ABA receptor, ABA-RELATED/H SUBUNIT OF THE MAGNESIUM-PROTOPORPHYRIN IX CHELATASE/ GENOMES UNCOUPLED 5 (ABAR/CHLH/GUN5), underlies the circadian gating of ABA-regulated processes, including stomatal opening and drought tolerance^17^,^23^,^24^. In addition, the clock component PRR7 massively regulates drought- and ABA-related genes in a circadian-dependent manner^25^,^26^.

Despite intensive studies, the molecular mechanisms responsible for the reciprocal regulations between the circadian and ABA signaling pathways remain to be fully elucidated. Here, we report that an ABA-inducible R2R3-type MYB transcription factor, MYB96, forms a complex signaling network with core clock components. MYB96 binds directly to the *TOC1* promoter to activate its expression. TOC1 in turn regulates *MYB96* expression possibly via CCA1. The trio defines a novel feedback network that links ABA responses with the circadian clock. These regulatory loops provide a mechanism whereby the timing for maximal responsiveness to ABA is controlled to optimize plant fitness. The results position MYB96 at the center of the crosstalk between the circadian clock and ABA signaling to ensure gated ABA responses at specific time-of-day.

## Results

### The expression of *MYB96* is under the control of the circadian clock

**Figure 1:**
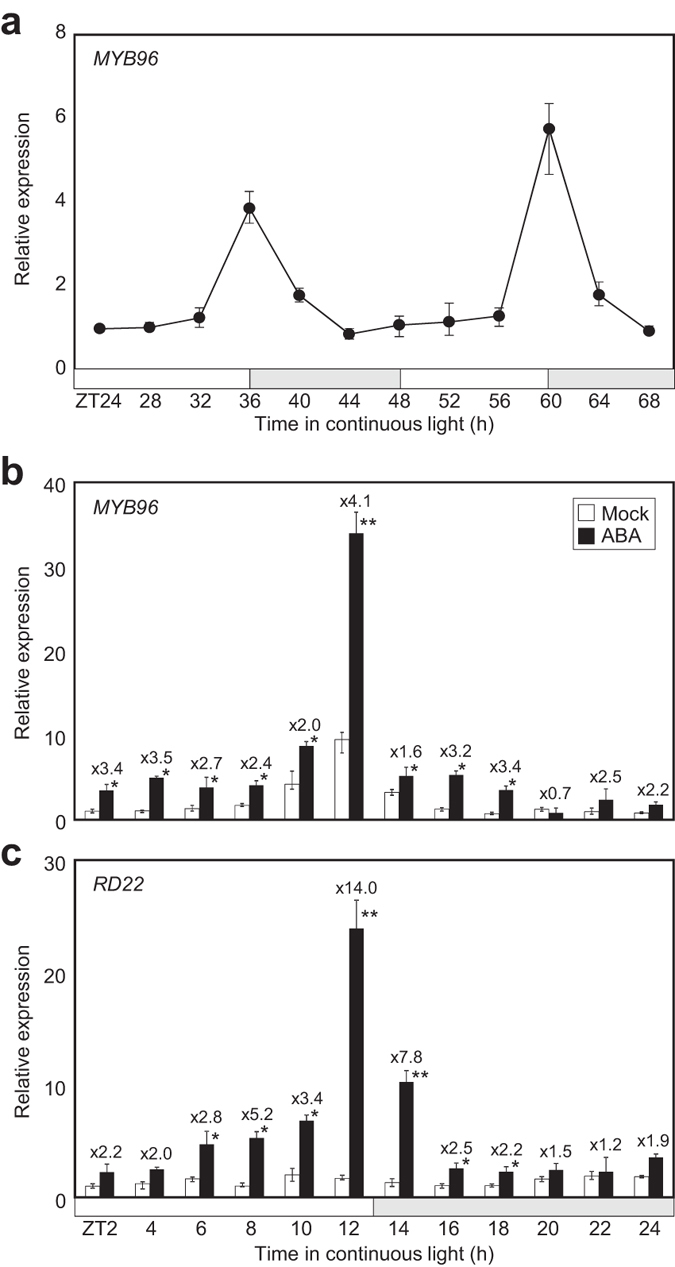
Circadian clock regulation of *MYB96* expression. (**a**) Circadian expression of *MYB96*. Seedlings grown under neutral day conditions (ND) for 10 days were transferred to continuous light conditions (LL) at Zeitgeber Time 0 (ZT0). Transcript levels were determined by quantitative real-time RT-PCR (RT-qPCR). Gene expression values were normalized to the *EUKARYOTIC TRANSLATION INITIATION FACTOR 4A1* (*eIF4A*) expression and represented as *n*-fold compared to the value of the sample at ZT24. Biological triplicates were averaged. Bars represent the standard error of the mean. (**b**,**c**) Circadian gating of ABA induction of *MYB96* (**b**) and *RD22* (**c**). Ten-day-old seedlings grown under ND were transferred to MS-liquid medium supplemented with or without 20 μM ABA for 2 h under LL and harvested at the indicated ZT points. Gene expression values were normalized to the *eIF4A* expression and represented as *n*-fold compared to the value of the mock-treated wild-type sample at ZT2. Three biological replicates were averaged and statistically significant differences between mock and ABA values are indicated by asterisks (Student’s *t*-test, **P < 0.01; *P < 0.05). Bars indicate standard error of the mean. The numbers above the bars indicate the ratio of expression in mock and ABA-treated samples (ABA/mock). The white and pale grey boxes indicate the subjective day and night, respectively.

The MYB96 transcription factor is a pivotal ABA signaling mediator in a variety of physiological processes, such as lateral root development, hormone homeostasis, carbon metabolism, and cuticular wax biosynthesis, and contributes to enhancing plant fitness under adverse environmental conditions^27^,^28^,^29^. Considering the close relationship between the clock and stress responses^30^,^31^,^32^, we speculated that MYB96 could be associated with clock activity. To examine this hypothesis, we analyzed the circadian expression of *MYB96* in wild-type (WT) seedlings entrained under neutral day conditions (ND; 12-h light/12-h dark) for 10 days and transferred to continuous light conditions (LL) for 3 days. Quantitative real-time RT-PCR (RT-qPCR) analysis revealed a rhythmic expression of *MYB96* that peaked around subjective dusk (Fig. 1a).

The *MYB96* gene is responsive to high levels of ABA^27^. Since ABA biosynthesis is also controlled by the circadian clock^14^, we asked whether circadian expression of *MYB96* was dependent on the oscillating endogenous ABA. We employed the ABA-deficient *aba3-1* mutant and analyzed the circadian expression of *MYB96* in the mutant. Circadian oscillation of *MYB96* was still observed in the *aba3-1* mutant (Supplementary Fig. S1), implying that the circadian clock directly regulates *MYB96* expression under normal growth conditions, rather than through the control of ABA metabolism.

As *MYB96* expression was regulated by the circadian clock (Fig. 1a), we postulated that *MYB96* induction by ABA might be also gated by the clock. To examine this possibility, we applied ABA to wild-type seedlings for 2 hours at different times during the day and analyzed the expression of *MYB96* and its downstream gene *RESPONSIVE TO DESSICATION 22* (*RD22*), whose expression correlates with MYB96 activity in an ABA-dependent signaling pathway^27^. Interestingly, *MYB96* and *RD22* were highly induced around Zeitgeber Time (ZT) 12 compared with other time points (Fig. 1b,c), consistent with the circadian expression pattern of *MYB96*. Together, our observations suggest that the circadian clock controls *MYB96* rhythmic expression and its ABA-dependent gated induction.

### MYB96 binds to the *TOC1* promoter

**Figure 2:**
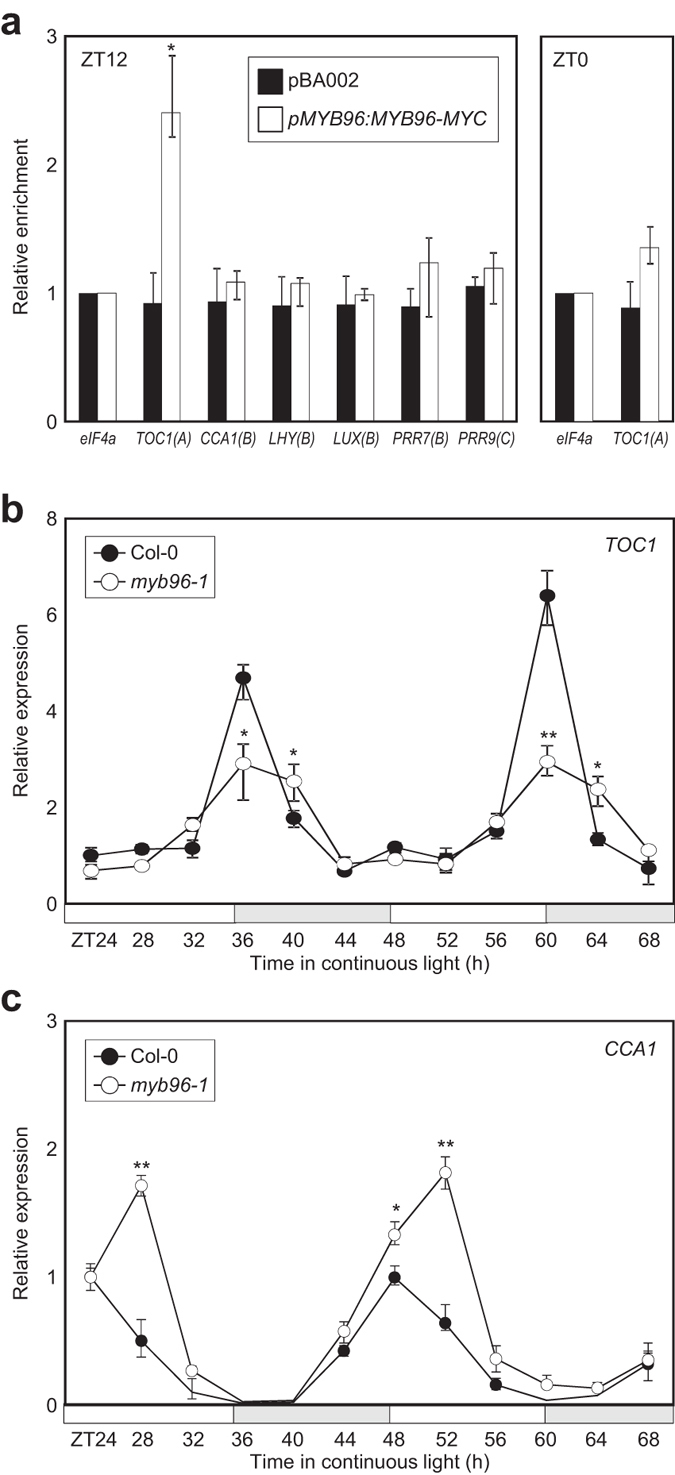
MYB96 regulation of *TOC1* expression and binding to the *TOC1* promoter. (**a**) ChIP assays showing the enrichment of putative R2R3-MYB binding regions in promoters of core clock genes analyzed by ChIP-PCR. Seedlings grown under ND were transferred to LL at ZT0. The regions for PCR amplification were shown in Supplementary Figure S2. Biological triplicates were averaged and statistical significance of the measurements was determined by a Student’s *t-*test (*P < 0.05). Bars indicate the standard error of the mean. (**b**) Expression of *TOC1* in *myb96-1*. (**c**) Expression of *CCA1* in *myb96-1*. In (**b,c**), seedlings grown under ND were transferred to LL at ZT0. Whole seedlings were harvested from ZT24 to ZT68 to analyze transcript accumulation. Gene expression values were normalized to the *eIF4A* expression and represented as *n*-fold compared to the value of the wild-type sample at ZT24. Biological triplicates were averaged (Student’s *t*-test, **P < 0.01; *P < 0.05; difference between wild-type and *myb96-1* plants). Bars indicate the standard error of the mean. The white and pale grey boxes indicate the subjective day and night, respectively.

As core clock genes contain conserved R2R3-type MYB-binding sequence motifs in their promoters, we hypothesized that MYB96 may be involved in the control of the clock activity. Therefore, we investigated whether MYB96 could bind to the promoters of core clock components such as *CCA1*, *LHY*, *LUX*, *TOC1*, *PRR7*, and *PRR9*. We conducted chromatin immunoprecipitation (ChIP) assays using *pMYB96:MYB96-MYC* transgenic plants, and DNA enrichment by ChIP was analyzed by qPCR (Supplementary Fig. S2). The analysis showed no significant amplification except for the specific binding to the *TOC1* promoter (Fig. 2a and Supplementary Fig. S2). Binding seems to occur mainly at ZT12 as no amplification was observed at other time point examined (Fig. 2a). Negative controls like immunoprecipitation with resin and without the antibody did not show a significant enrichment (Supplementary Fig. S3).

Binding of MYB96 at its peak phase of expression, which coincides with that of *TOC1* (Fig. 1a)^33^,^34^, suggests a positive regulation of *TOC1* by MYB96. Thus, we analyzed endogenous circadian expression of *TOC1* in *MYB96*-deficient *myb96-1* seedlings^27^ entrained under ND and subsequently transferred to LL. Our results showed that the rhythmic amplitude of *TOC1* expression was significantly reduced in *myb96-1* mutant particularly at *TOC1* peak of expression (Fig. 2b). To support a role of MYB96 in *TOC1* activation, we also analyzed *CCA1* expression, which is negatively regulated by TOC1, in *myb96-1* mutant plants. RT-qPCR analysis revealed higher amplitude and a delayed phase of *CCA1* expression in *myb96-1* compared to WT (Fig. 2c). Our results suggest that MYB96 might indirectly regulate *CCA1* expression by modulating *TOC1* expression and/or other related components.

### MYB96 regulates ABA gated induction of *TOC1*

**Figure 3:**
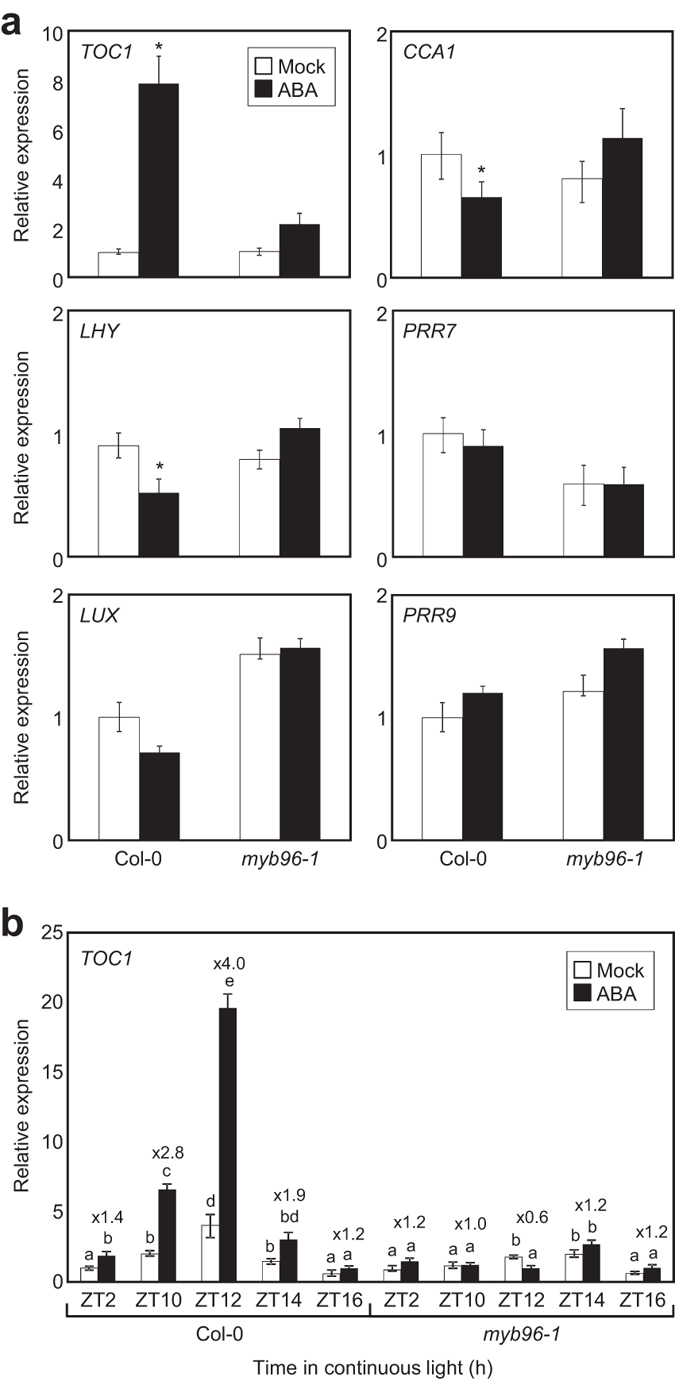
Regulation of *TOC1* circadian gating by MYB96. (**a**) Induction of *TOC1* by ABA. Ten-day-old seedlings grown under ND conditions were transferred to MS-liquid medium supplemented with or without 20 μM ABA at ZT8 and incubated for 2 h under LL. Transcript accumulation was analyzed by RT-qPCR. Gene expression values were normalized to the *eIF4A* expression and represented as *n*-fold compared to the value of the mock-treated wild-type sample. Biological triplicates were averaged (Student’s *t*-test, *P < 0.05). Bars indicate the standard error of the mean. (**b**) Time course analysis of *TOC1*circadian gating in *myb96-1*. Gene expression values were normalized to the *eIF4A* expression and represented as *n*-fold compared to the value of the mock-treated wild-type sample at ZT2. Three biological replicates were averaged. Different letters represent a significant difference at *P* < 0.05 (one-way anova with Fisher’s *post hoc* test). Bars indicate standard error of the mean. The numbers above the bars indicate the ratio of expression in mock and ABA-treated samples (ABA/mock).

Given the implication of MYB96 in both ABA signaling^27^,^28^,^29^ and clock gene regulation, MYB96 is likely to have a role on ABA regulation of clock activity. To explore this notion, we examined ABA responsiveness of core clock genes in wild-type and *myb96-1* mutant plants. Consistent with previous results^17^, *TOC1* expression was substantially induced by exogenous ABA before and around dusk, a time when *MYB96* is highly expressed (Fig. 3a). Notably, the ABA gated induction of *TOC1* was fully dependent on MYB96 function, as *TOC1* induction by ABA was abolished in *myb96-1* mutant plants (Fig. 3a). In *myb96-1* mutant plants, *TOC1* was not significantly induced by ABA at other time points examined, which excludes the possibility that the circadian phase phenotype of *myb96-1* mutant plants was responsible for a different timing of *TOC1* induction (Fig. 3b). Our results also showed that expression of *CCA1* and *LHY* was slightly suppressed by ABA, which may be a result of *TOC1* activation, in a MYB96-dependent manner (Fig. 3a). Together, the results suggest that MYB96 has a crucial role mediating the gated induction of *TOC1* by ABA.

**Figure 4:**
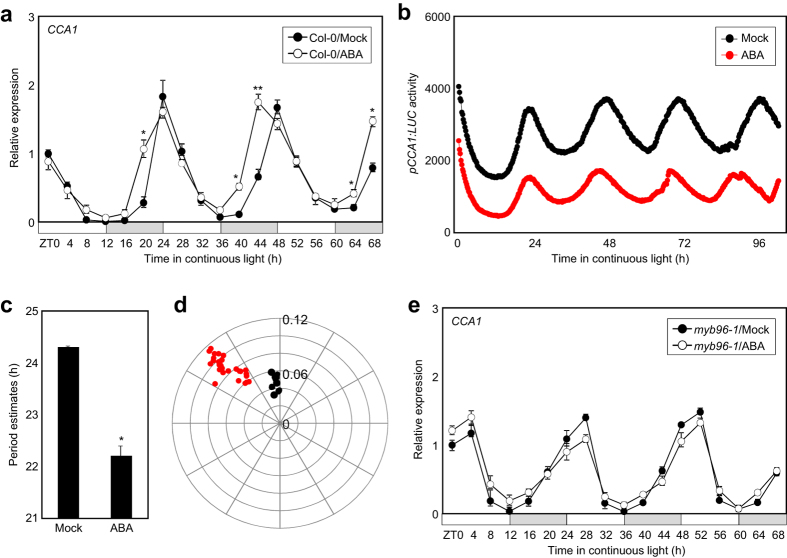
Role of MYB96 regulating the changes on ABA-mediated rhythms. (**a**) Effects of ABA on *CCA1* expression in wild-type plants. (**b**) Analysis of *pCCA1:LUC* circadian activity. Ten-day-old *pCCA1:LUC* transgenic plants grown under ND were transferred to LL. Luciferase activities were examined in the presence or absence of 20 μM ABA that was added at ZT0. Waveforms represent the average of 42 plants for each condition. (**c**) Period estimates of *pCCA1:LUC* activity in the presence or absence of 20 μM ABA. Bars indicate the standard error of the mean (*P < 0.05; Student’s *t*-test). (**d**) Phase plot of *pCCA1:LUC* activity in the presence or absence of 20 μM ABA. Phases were plotted against the strength of the rhythm expressed as relative amplitude error. The rhythm strength is graphed from 0 (center of the plot) to 0.12 (periphery of the circle). (**e**) Effects of ABA on *CCA1* expression in *myb96-1*. In (**a**,**e**), ten-day-old seedlings grown under ND were transferred to LL in the absence or presence of 20 μM ABA. Transcript accumulation was analyzed by RT-qPCR. Gene expression values were normalized to the *eIF4A* expression and represented as *n*-fold compared to the value of the mock-treated wild-type sample at ZT0 (**a**). Biological triplicates were averaged (Student’s *t*-test, **P < 0.01; *P < 0.05; difference between ABA-treated and mock-treated plants). Bars indicate the standard error of the mean. The white and pale grey boxes indicate the subjective day and night, respectively.

It has been previously shown that ABA affects the circadian phase and period length^26^. In our growth conditions, an advanced phase of *CCA1* expression was observed in wild-type seedlings treated with ABA (Fig. 4a). The use of plants expressing the *CCA1* promoter fused to the *LUCIFERASE* (*pCCA1:LUC*) also revealed the advanced phase and a short circadian period in samples treated with ABA (circadian period without ABA: Mean = 24.31; SEM = 0.01; SD = 0.050; versus circadian period with ABA: Mean = 22.21; SEM = 0.03; SD = 0.21; *p*-value < 0.05) (Fig. 4b–d). In contrast, the *myb96-1* mutant was insensitive to ABA and no observable differences in expression were observed after ABA treatment (Fig. 4e). While the *myb96-1* mutant plants showed altered rhythmic oscillation possibly due to the disruption of circadian homeostasis, ABA did not further influence the circadian rhythms (Fig. 4e), demonstrating that MYB96 is a crucial regulator that relays ABA signaling to the clock circuitry.

### Expression of *MYB96* is influenced by TOC1

**Figure 5:**
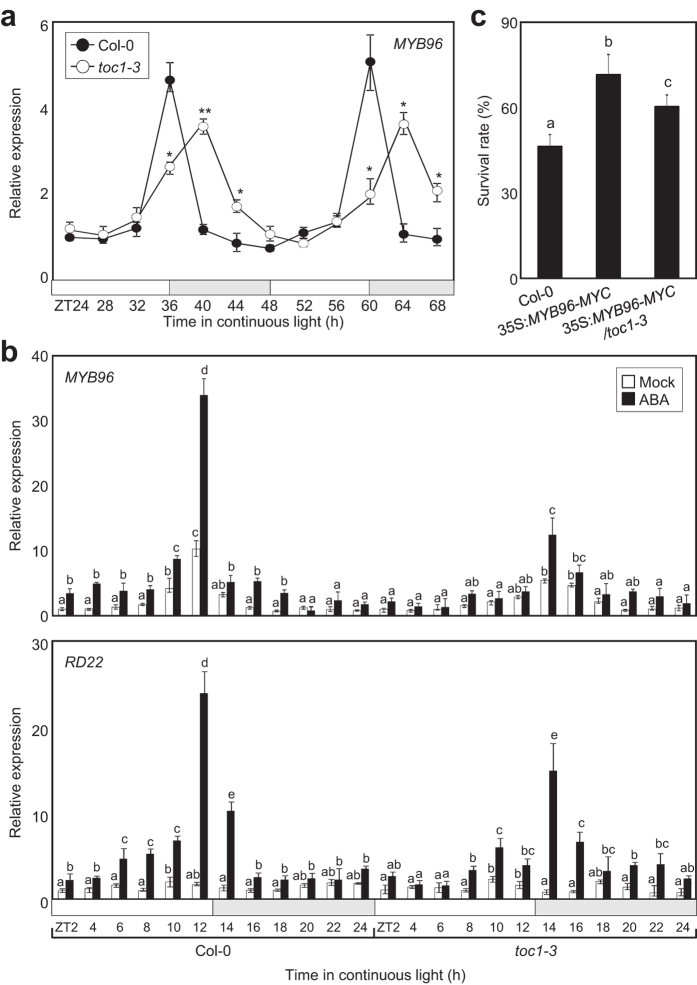
Regulation of *MYB96* by TOC1. (**a**) Time course analysis of *MYB96* expression in *toc1-3*. Student’s *t*-test, **P < 0.01; *P < 0.05; difference between wild-type and *toc1-3* plants. (**b**) Disruption of circadian gating of *MYB96* (upper panel) and *RD22* (lower panel) in *toc1-3*. Seedlings grown under ND conditions for 10 days were transferred to LL. Gene expression values were normalized to the *eIF4A* expression and represented as *n*-fold compared to the value of the mock-treated wild-type sample at ZT2. Three biological replicates were averaged. Different letters represent a significant difference at *P* < 0.05 (one-way anova with Fisher’s *post hoc* test). Bars indicate standard error of the mean. The white and pale grey boxes indicate the subjective day and night, respectively. (**c**) Analysis of plant survival to drought conditions. Two-week-old plants were subjected to drought conditions by withholding water for two weeks. Plant survival rate was determined 3 d after rewatering. Biological triplicates were averaged. Different letters represent a significant difference at *P* < 0.05 (one-way anova with Fisher’s *post hoc* test). Bars indicate standard error of the mean.

Due to the important role of TOC1 on ABA-mediated responses, it may be possible that TOC1 establishes a feedback regulation with MYB96. To examine this possibility, we used the *toc1-3* mutant plants (Columbia-0 background), which lack *TOC1* transcript accumulation and show circadian and morphological phenotypes similar to those described for *toc1-1* (Supplementary Fig. S4). RT-qPCR analysis of *MYB96* expression revealed a phase delay and slightly decreased amplitude of *MYB96* expression in *toc1-1* and *toc1-3* mutants (Fig. 5a and Supplementary Fig. S5), which suggest that a functional TOC1 is necessary for proper *MYB96* rhythmic expression.

We also examined *MYB96* and *RD22* expression in WT and *toc1-3* mutant plants treated with ABA. Our results showed that ABA induction of *MYB96* and *RD22* was significantly reduced and their peak expression was shifted to ZT14 (and ZT38) in *toc1-3* (Fig. 5b and Supplementary Fig. S6), which supports the requirement of TOC1 for proper ABA-mediated induction of *MYB96*. It is noteworthy that *toc1-3* (Supplementary Fig. S4) and *toc1-1*^3^ usually advance the phase of circadian clock genes but in the case of *MYB96* and *RD22*, the ABA-mediated induction was delayed rather than advanced (Fig. 5b and Supplementary Fig. S6). These results might be indicating that TOC1 controls the ABA gated induction of *MYB96* and *RD22* through mechanisms both dependent and independent of the clock.

To examine the biological relevance of MYB96 and TOC1 relationship, we genetically crossed *MYB96*-overexpressing transgenic plants (35S:*MYB96-MYC*) with *toc1-3* mutant and analyzed the susceptibility to drought. Our results showed that the enhanced tolerance to drought of 35S:*MYB96-MYC* plants was slightly but significantly suppressed by the *toc1-3* mutation (Fig. 5c). Consistently, the higher expression of stress-responsive genes in 35S:*MYB96-MYC* was also compromised in 35S:*MYB96-MYC toc1-3* plants (Supplementary Fig. S7). These results reinforce the drought phenotypes and suggest that indeed the function of MYB96 on drought tolerance requires the presence of a functional TOC1.

### CCA1 might also regulate the MYB96-TOC1 module

Our results suggest that the MYB96-TOC1 module is relevant for the circadian gating of ABA signaling in *Arabidopsis*. However, it is unclear how TOC1 affects *MYB96* expression. Given the biochemical nature of TOC1 as a transcriptional repressor, it is unlikely that TOC1 binds directly to the *MYB96* promoter to stimulate its expression. To explore the circadian molecular component responsible for the regulation of *MYB96*, we performed analysis of the *cis*-elements present within the *MYB96* promoter. Multiple CCA1-binding sites (CBSs, AAAATCT) and evening elements (EEs, AAATATCT) were found in the promoter (Supplementary Fig. S8). The presence of the elements led us to examine whether CCA1 could bind to the *MYB96* promoter. We performed ChIP assays using plants expressing *CCA1* under its own promoter (*pCCA1:CCA1-HA-YFP cca1-1*)^35^. Total protein extracts of samples collected at ZT0 were immunoprecipitated with anti-HA antibody. DNA bound to epitope-tagged CCA1 proteins was analyzed by qPCR analysis. The C and D regions of the *MYB96* promoter containing CBS elements were enriched in the ChIP experiments (Supplementary Fig. S8). Binding of CCA1 to the *MYB96* promoter was specifically observed at dawn but not at dusk (Supplementary Fig. S8). To further support the regulation of *MYB96* by CCA1, we analyzed *MYB96* expression in *cca1-2* mutant plants^30^. Our results revealed that the peak phase of *MYB96* expression was advanced in the *cca1-2* mutant compared to WT plants, with an overall decreased amplitude but a slightly increased expression around dawn (e.g. ZT48–52) (Supplementary Fig. S9). Thus, TOC1 may indirectly bolster *MYB96* expression by its repression of *CCA1*. These results support a complex feedback network of direct and indirect regulations in the control of *CCA1*, *MYB96* and *TOC1* expression.

Taken together, our results suggest that the MYB96 transcription factor, a pivotal regulator of ABA signaling, is connected with the core of the clock. MYB96 function not only exemplifies the bidirectional regulation of the circadian clock and ABA signaling, but also contributes to gating of ABA responses. This signaling scheme explains the acute ABA responses at specific times during the day that facilitate a proper balance of plant growth and energy-consuming stress responses.

## Discussion

### Circadian gating of ABA signaling

A significant fraction of genes involved in ABA metabolism, perception and signaling are regulated by the circadian clock^13^,^14^,^25^,^36^. Efficient ABA responses are required to ensure a perfect balance between water loss and photosynthesis in the day^23^,^24^,^25^. In this study, we demonstrate that the crucial ABA signaling factor MYB96 is connected with core clock components, forming extensive circadian networks with direct and indirect regulations. The linkage of MYB96 with clock components possibly shapes the circadian gating of ABA responses. The circadian clock defines the diurnal ABA induction of *MYB96* and its downstream genes that peak around dusk. Accordingly, mis-regulation of *TOC1* leads to the disruption of ABA-mediated induction of *MYB96* and MYB96-mediated ABA signaling. In addition, MYB96 positively regulates *TOC1*, which in turn stimulates *MYB96* expression to facilitate its time-of-day-dependent activation. The mutual synergistic activation of the MYB96-TOC1 module might ensure robust temporal regulation of ABA signaling.

Although CCA1 plays a role modulating the transcript accumulation of *MYB96* by binding to its promoter, the full set of clock factor(s) that directly regulates the circadian gating of *MYB96* remains to be further elucidated. Further studies are also required to unravel the complete molecular mechanism underlying the bidirectional regulation, but the intersection of MYB96 with the clock oscillator is important for maximal responses to ABA.

The circadian clock precisely regulates essential physiological processes to occur at specific times of day^37^. Coordination of endogenous physiology to external environmental cycles not only maximizes metabolic efficiency, but also establishes an efficient way to increase stress tolerance without substantially decreasing plant growth. Most likely, our results with MYB96 are not an exception and a considerable number of stress signaling factors may be associated with clock components to achieve acute gating of endogenous stress responses.

### ABA regulation of circadian clock

In addition to the circadian regulation of ABA responses, ABA signaling reciprocally regulates clock activity^26^,^38^,^39^,^40^. This bidirectional regulation might enable plants to timely gate physiological and molecular responses under normal conditions but also trigger stress responses to unexpected environmental challenges.

MYB96 is likely a molecular component responsible for the bidirectional regulation between ABA and the clock. Besides the regulation of *MYB96* by the circadian clock, the MYB96 protein subsequently influences clock activity possibly via TOC1. The circadian network built up from MYB96 is particularly relevant when ABA is applied. In the presence of ABA, MYB96 activation of *TOC1* probably suppresses *CCA1* expression. When ABA is applied at certain times of day, other than dusk, the ABA-activated MYB96-TOC1 module suppresses CCA1 activity in order to synchronize the circadian oscillation to the environmental signal. Indeed, ABA applied at ZT0, when high amplitude of *CCA1* expression is observed, shortens noticeably the circadian period (Fig. 4), which may be explained by the reduced activity of CCA1 that results in a shortened circadian period. The somehow controversial effects of ABA on the circadian clock might be due to the timing of treatment^26^,^38^,^39^,^40^. Alternatively, because carbon source is an important entrainment signal, the presence or absence of sucrose in the growth media might influence the sensitivity to ABA. Given that ABA-reduction of circadian period largely depends on MYB96, the MYB96 protein acts as a possible regulator mediating the interaction between the circadian clock and ABA signaling.

Together, our results provide a striking example of the intricate connections between clock components and stress signaling that improve the plant’s ability to anticipate environmental changes. This study also sheds light on the interaction between plants and the surrounding environment for their efficient environmental adaptation.

## Methods

### Plant materials and growth conditions

*Arabidopsis thaliana* (Columbia-0 ecotype) was used for all experiments, unless otherwise specified. Plants were grown under neutral day conditions (NDs; 12-h light/12-h dark cycles) with cool white fluorescent light (100 μmol photons m^−2^ s^−1^) at 23 °C. The *myb96-1* mutant (GABI_120B05) and 35S:*MYB96-MYC* and *pMYB96:MYB96-MYC* transgenic plants were previously reported^27^,^28^,^29^. The *CCA1*-deficient *cca1-2*^30^ and *pCCA1:CCA1-HA-YFP cca1-1*^35^ seeds were also previously described. The *toc1-3* (SALK_203853) and *aba3-1* (CS157) mutants were obtained from ABRC (*Arabidopsis* Biological Resource Center at the Ohio State University), and the lack of *TOC1* expression was confirmed by semi-quantitative RT-PCR.

A MYC-coding sequence was fused in frame to the 3′ end of the *MYB96* gene, and the gene fusion was subcloned under the CaMV 35S or endogenous promoter in the modified pBA002 vector^30^. The empty vector and expression constructs were transformed into Col-0 plants.

### Quantitative real-time RT-PCR analysis

Total RNA was extracted using TRI agent (TAKARA Bio, Singa, Japan) according to the manufacturer’s recommendations. Reverse transcription (RT) was performed using Moloney Murine Leukemia Virus (M-MLV) reverse transcriptase (Dr. Protein, Seoul, South Korea) with oligo(dT18) to synthesize first-strand cDNA from 2 μg of total RNA. Total RNA samples were pretreated with an RNAse-free DNAse. cDNAs were diluted to 100 μL with TE buffer, and 1 μL of diluted cDNA was used for PCR amplification.

Quantitative RT-PCR reactions were performed in 96-well blocks using the Step-One Plus Real-Time PCR System (Applied Biosystems). The PCR primers used are listed in Supplementary Table S1. The values for each set of primers were normalized relative to the *EUKARYOTIC TRANSLATION INITIATION FACTOR 4A1* (*eIF4A*) gene (At3g13920) as an internal control in each sample. Normalized values were represented relative to the control in each case. Values were represented as means ± SEM (standard error of the mean). All RT-qPCR reactions were performed in technical triplicates using total RNA samples extracted from three independent replicate samples. The comparative ΔΔC_T_ method was employed to evaluate relative quantities of each amplified product in the samples. The threshold cycle (C_T_) was automatically determined for each reaction by the system set with default parameters. The specificity of the RT-qPCR reactions was determined by melt curve analysis of the amplified products using standard methods. Differences in expression ratios (dCt) among the samples were analyzed by ANOVA or *t*-test.

### Treatments with ABA in seedlings

For treatment with ABA, 10-day-old seedlings grown under NDs were transferred to half-strength MS-liquid medium supplemented with 20 μM (+)-*cis*,*trans*-ABA (L06278) (Alfa Aesar, Ward Hill, MA, USA).

### Luminescence assays

Ten-day-old *CCA1:LUC*-expressing seedlings entrained under NDs were transferred into 96-well microplates containing sugar-free MS-liquid medium supplemented with 20 μM ABA and 60 μM D-luciferin (7092–100; Biovision, San Francisco, USA) at the ZT0 point. The luminescence rhythms were monitored using a microplate luminometer CentroXS^3^ LB 960 (Berthold Technologies, Wildbad, Germany) and analyzed as previously described^41^. Circadian period estimates were determined using the Biological Rhythms Analysis Software System (BRASS).

### Drought tolerance assays

Two-week-old plants were subjected to drought condition by withholding water for two weeks. To prevent direct air drying of seedlings, small pores were made in the plastic cover 7 d following the start of drought, and the cover was removed 7 d later. Plant survival rate was determined 3 d after rewatering. At least five containers of three genotypes (30 plants/container) were evaluated in three independent experiments.

### Chromatin immunoprecipitation

Chromatin immunoprecipitation (ChIP) assays were performed as previously described^42^. The epitope-tagged transgenic plants, corresponding antibodies (Millipore, Billerica, USA) and salmon sperm DNA/protein A agarose beads (Millipore, Billerica, USA) were used for ChIP. DNA was purified using phenol/chloroform/isoamyl alcohol and sodium acetate (pH 5.2). The level of precipitated DNA fragments was quantified by quantitative real-time PCR using specific primer sets (Supplementary Table S2). The values were normalized with the input DNA level. The values in control plants were set to 1 after normalization against *eIF4a* for quantitative PCR analysis.

## Additional Information

**How to cite this article**: Lee, H.G. *et al.* MYB96 shapes the circadian gating of ABA signaling in *Arabidopsis*. *Sci. Rep.* **6**, 17754; doi: 10.1038/srep17754 (2016).

## Supplementary Material

Supplementary Information
