# Supplementary material for: MYB96 shapes the circadian gating of ABA signaling in Arabidopsis
Source: Sci Rep. 2016 Jan 4;6:17754. doi: 10.1038/srep17754 (PMC4698719; doi:10.1038/srep17754)
Supplement: Supplementary Information [file srep17754-s1.pdf]

SUPPLEMENTARY INFORMATION

MYB96 shapes the circadian gating of ABA signaling in *Arabidopsis*

Hong Gil Lee, Paloma Mas & Pil Joon Seo

Supplementary Figures

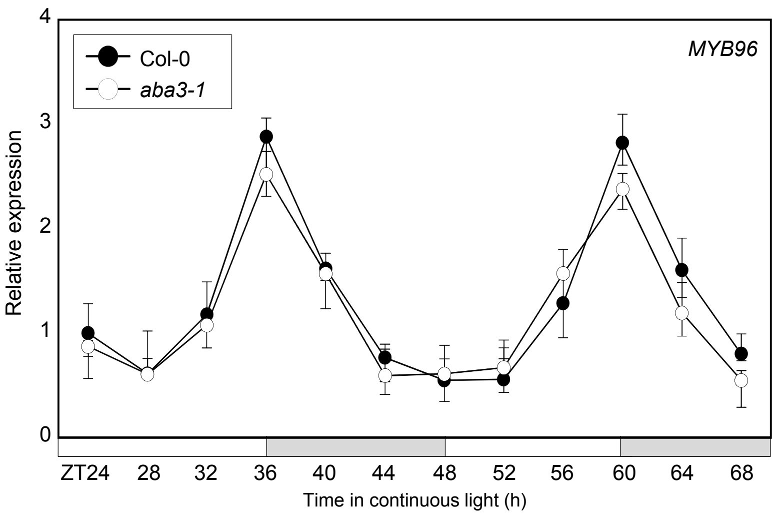

Supplementary Figure S1. Circadian expression of *MYB96* in *aba3-1*.
Seedlings grown under neutral day conditions (ND; 12-h light/12-h dark) for 10 days were transferred to continuous light conditions (LL) at Zeitgeber Time 0 (ZT0). Seedlings were harvested from ZT24 to ZT68. Transcript levels were determined by quantitative real-time RT-PCR (RT-qPCR). Biological triplicates were averaged. Bars represent the standard error of the mean. The white and pale grey boxes indicate the subjective day and night, respectively.

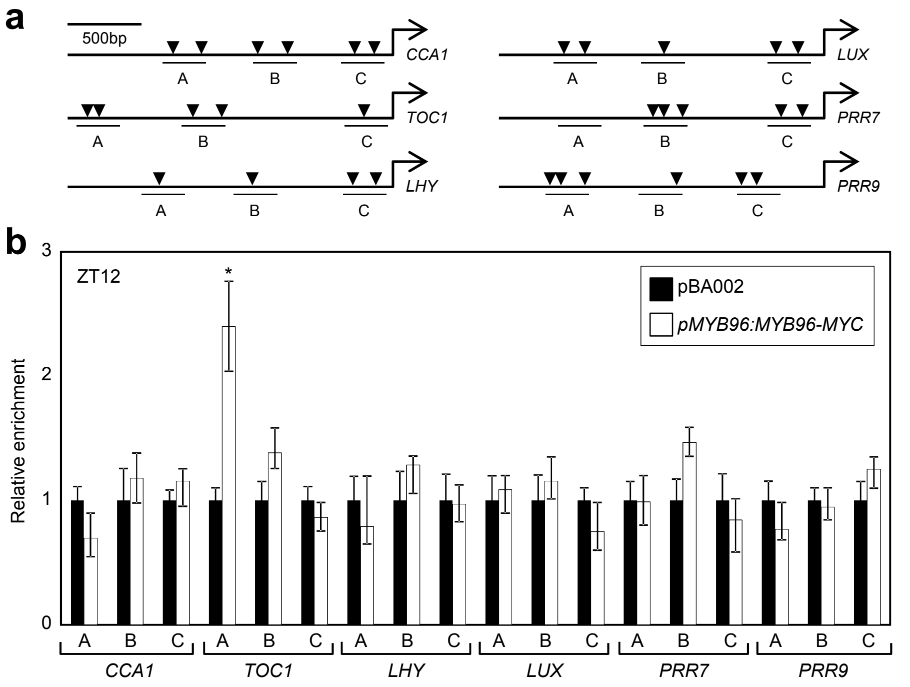

Supplementary Figure S2. Chromatin immunoprecipitation (ChIP) assays.

(**a**) Promoter analysis of core clock genes. The putative R2R3-MYB binding sites are indicated by arrowheads. Black thin lines indicate the regions for PCR amplification after ChIP.

(**b**) Specific binding of MYB96 to the *TOC1* promoter. Enrichment of putative MYB binding regions of the core clock gene promoters was analyzed by ChIP-PCR. The values in pBA002 plants were set to 1 after normalization against eIF4a for qPCR analysis. Biological triplicates were averaged and statistically analyzed with Student's *t*-test (*P<0.05). Bars indicate standard error of the mean.

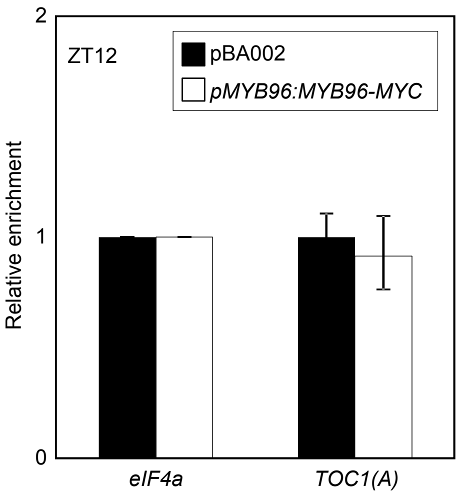

Supplementary Figure S3. ChIP assays using antibody-free resin.

Enrichment of the putative R2R3-MYB binding region of the *TOC1* promoter was analyzed by qPCR after ChIP with resin alone. Biological triplicates were averaged. Bars indicate standard error of the mean.

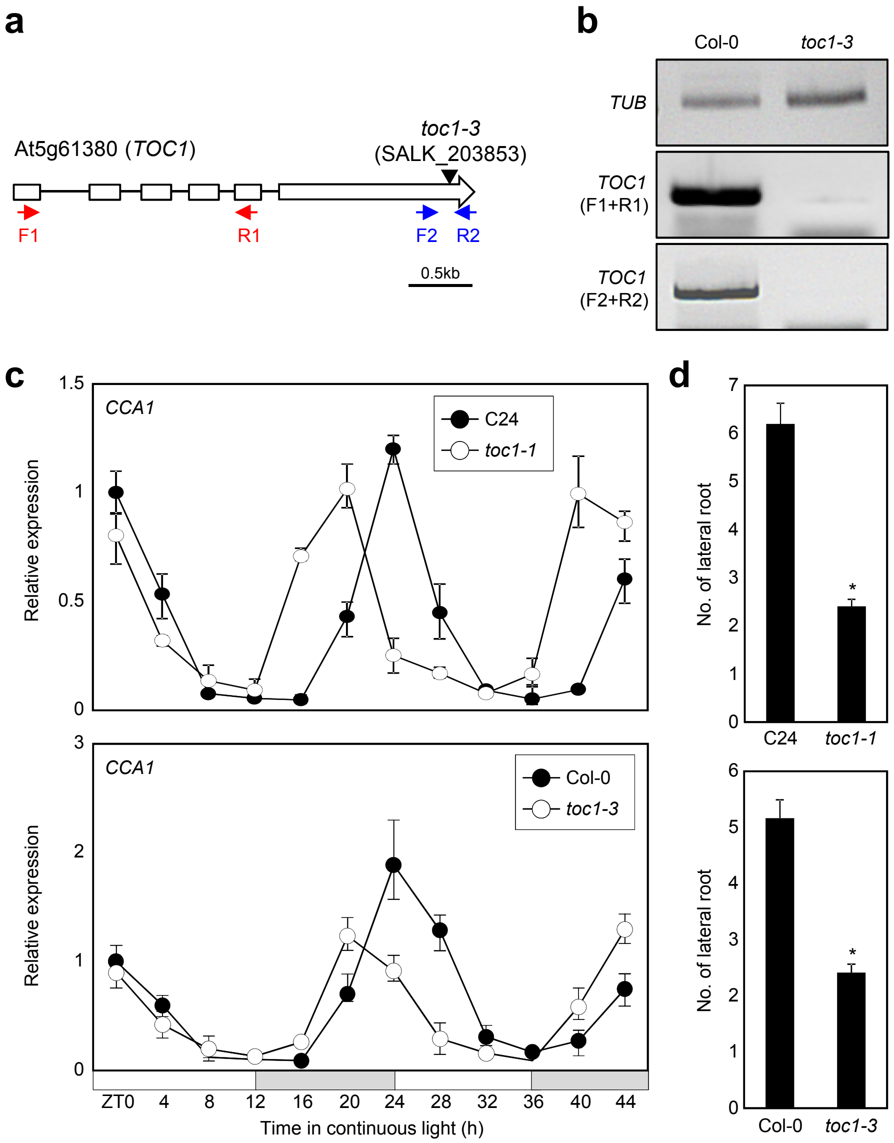

Supplementary Figure S4. Characterization of *toc1-3* mutant plants

(**a**) Schematic representation showing the T-DNA insertion site in *toc1-3* mutant. White bars indicate exons of the *TOC1* gene. The black arrowhead indicates the T-DNA insertion site. Colored arrows indicate the position of primers used for the analyses.

(**b**) Analysis of *TOC1* transcript accumulation in *toc1-3*. Transcript accumulation was analyzed by RT-PCR using the indicated primers. The *TUBULIN BETA CHAIN 2* (*TUB*) gene (At5g62690) was used as an internal control.

(**c**) *CCA1* expression in *toc1-1* (upper panel) and in *toc1-3* (lower panel). Ten-day-old seedlings grown under ND were transferred to LL. Transcript levels were determined by RT-qPCR. Biological triplicates were averaged. Bars represent the standard error of the mean. The white and pale grey boxes indicate the subjective day and night, respectively.

(**d**) Lateral root phenotypes. Ten-day-old seedlings were fixed and used to count emerged lateral root numbers. Biological triplicates were averaged. Bars represent the standard error of the mean (**P*<0.05; Student's *t*-test). Note that lateral root phenotypes of *toc1-3* were similar to those of *toc1-1*[1].

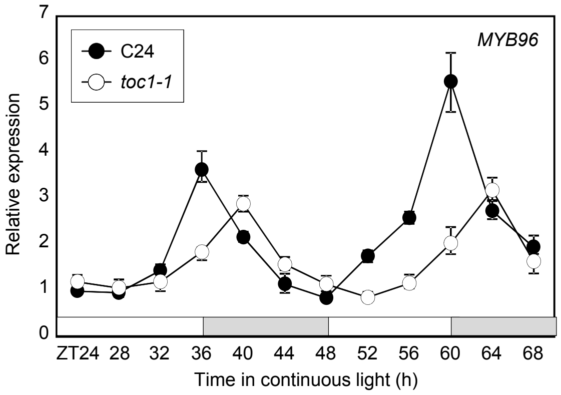

Supplementary Figure S5. Expression of *MYB96* in *toc1-1*.
Seedlings grown under ND conditions for 10 days were transferred to LL conditions at ZT0. Seedlings were harvested from ZT24 to ZT68. Transcript levels were determined by RT-qPCR. Biological triplicates were averaged. Bars represent the standard error of the mean. The white and pale grey boxes indicate the subjective day and night, respectively.

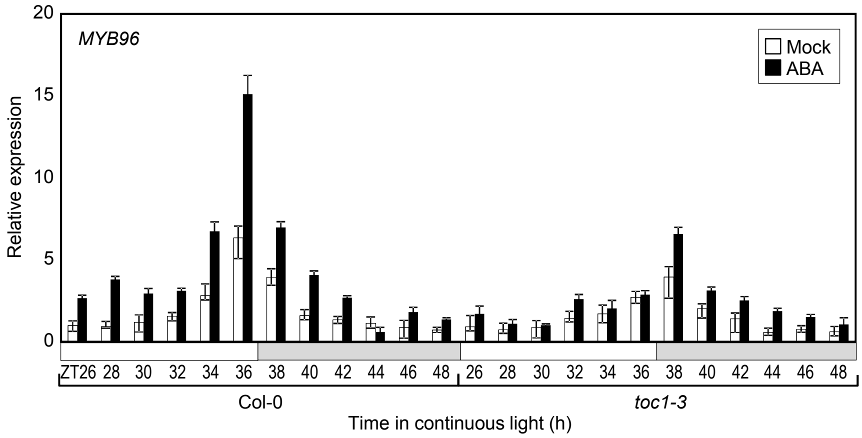

Supplementary Figure S6. ABA induction of *MYB96* in *toc1-3*.
Ten-day-old seedlings grown under ND were transferred to MS-liquid medium supplemented with or without 20 μM ABA for 2 h under LL and harvested at the indicated ZT points. Transcript levels were determined by RT-qPCR. Biological triplicates were averaged. Bars represent the standard error of the mean. The white and pale grey boxes indicate the subjective day and night, respectively.

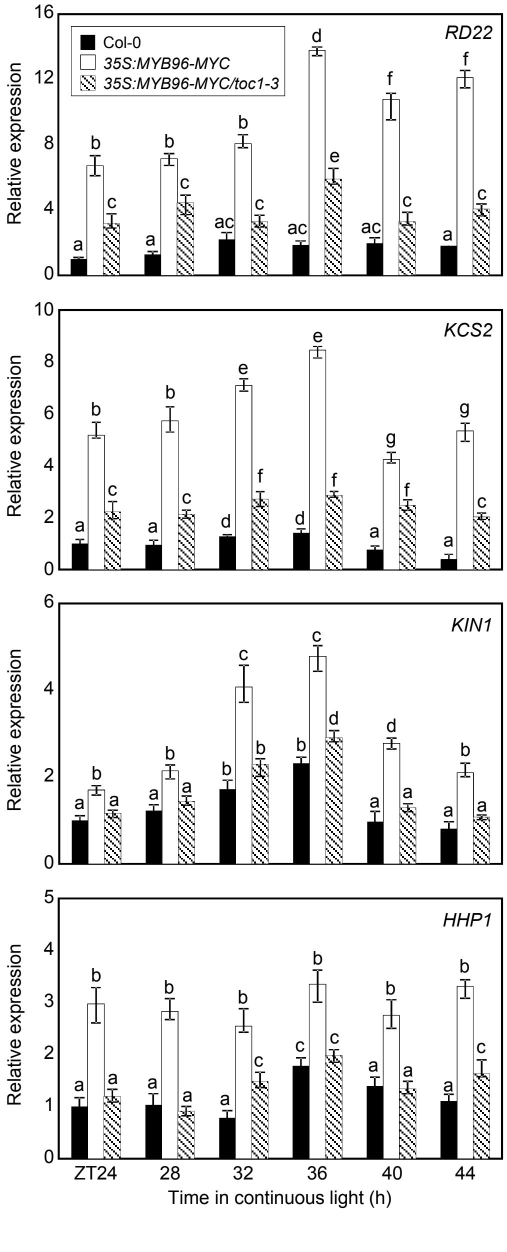

Supplementary Figure S7. Expression of stress-responsive genes.

Ten-day-old seedlings grown under ND conditions were harvested at indicated time points for total RNA isolation of the different genetic backgrounds. Transcript accumulation of stress-responsive genes regulated by MYB96[2-4] was analyzed by RT-qPCR. The *eIF4a* gene was used as an internal control. Biological triplicates were averaged. Different letters represent a significant difference at $P<0.05$ (one-way anova with Fisher's *post hoc* test). Bars indicate the standard error of the mean.

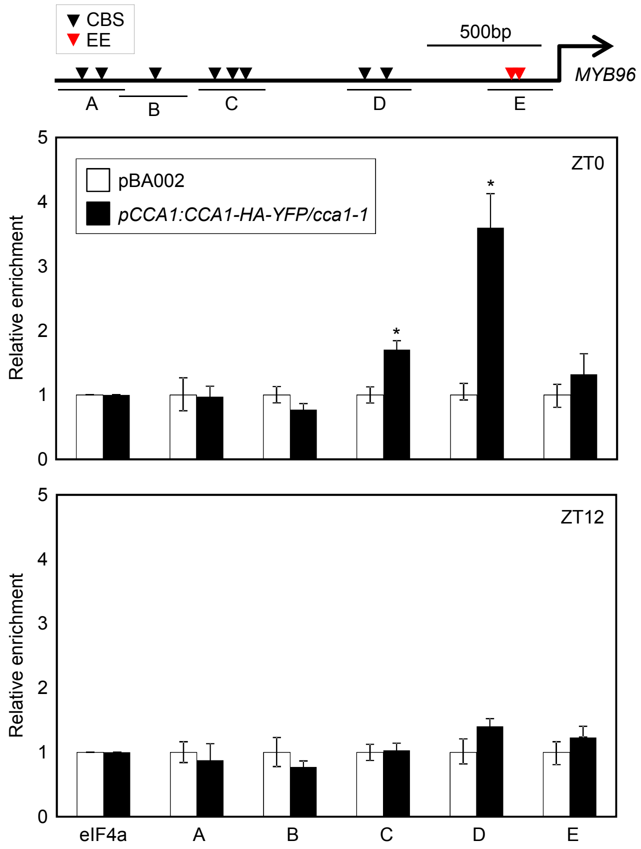

Supplementary Figure S8. CCA1 binding to the *MYB96* promoter.
Schematic representation of the *MYB96* promoter depicting the putative CCA1 binding sites (CBSs) and evening elements (EEs) (black and red arrowheads, respectively). Underbars indicate the regions for PCR amplification after the ChIP assays. Enrichment of the putative CCA1-binding regions in the *MYB96* promoter was analyzed by ChIP-PCR. Biological triplicates were averaged and statistical significance of the measurements was determined by a Student's *t*-test (**P*<0.05). Bars indicate the standard error of the mean.

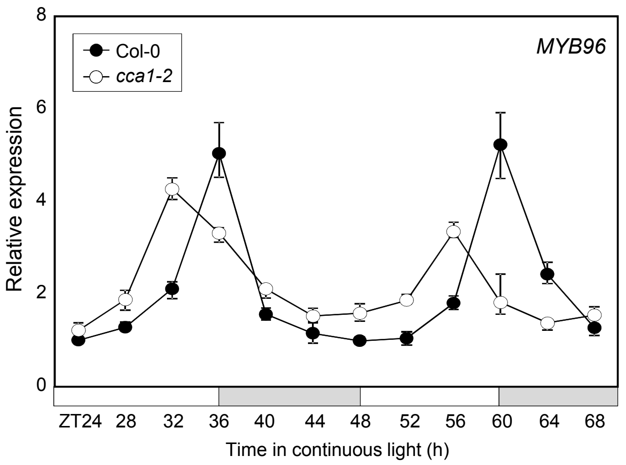

Supplementary Figure S9. ***MYB96*** **expression in** ***cca1-2*****.**
Seedlings grown under ND for 10 days were transferred to LL at ZT0. Transcript levels were determined by RT-qPCR. Biological triplicates were averaged. Bars represent the standard error of the mean. The white and pale grey boxes indicate the subjective day and night, respectively.

Supplementary Tables

Primer	Usage	Sequence
eIF4a-F	RT-qPCR	5′-TGACCACACAGTCTCTGCAA
eIF4a-R	RT-qPCR	5′-ACCAGGGAGACTTGTTGGAC
MYB96-F	RT-qPCR	5′-TGCAGTCTCGGAAGAAGGTG
MYB96-R	RT-qPCR	5′-CATCTCGTGGCTTTGCTCAT
CCA1-F	RT-qPCR	5′-GATCTGGTTATTAAGACTCGGAAGCCATATAC
CCA1-R	RT-qPCR	5′-GCCTCTTTCTCTACCTTGGAGA
TOC1-F	RT-qPCR	5′-TCTTCGCAGAATCCCTGTGAT
TOC1-R	RT-qPCR	5′-GCTGCACCTAGCTTCAAGCA
LHY-F	RT-qPCR	5′-CTTATCCTCGAAAGCCTGGG
LHY-R	RT-qPCR	5′-GAACATCTTGAACCGCGTTG
LUX-F	RT-qPCR	5′-GTTCCTCCACAGAGCTTCCA
LUX-R	RT-qPCR	5′-CTGAACAGATGCAATTTGGGA
PRR7-F	RT-qPCR	5′-TGGGCCATATGGAAGCAGTA
PRR7-R	RT-qPCR	5′-TTTACGCACAAATTGGCCTC
PRR9-F	RT-qPCR	5′-TTGGTCCTGAGCTTGGACTTT
PRR9-R	RT-qPCR	5′-GCTTACGCTTGATGATCCGA
RD22-F	RT-qPCR	5′-AGGAGCAAACCCTTTCGTGT
RD22-R	RT-qPCR	5′-CGTTTCAACGTCTCCGAAAA
KCS2-F	RT-qPCR	5′-CAACCTCGCTTTCCAACAAA
KCS2-R	RT-qPCR	5′-TCCGGTTTTCTCAAGCACTG
KIN1-F	RT-qPCR	5′-TGGAGCTGGAGCACAACA
KIN1-R	RT-qPCR	5′-GACCCGAATCGCTACTTGTTC
HHP1-F	RT-qPCR	5′-GCAAGCAGCATTTGTCACCT
HHP1-R	RT-qPCR	5′-TGCCTGCGAGGTAGATGAAG

Supplementary Table S1. Primers used in this study.
RT-qPCR primers were designed using the Primer Express Software installed into the Applied Biosystems 7500 Real-Time PCR System. The sizes of PCR products ranged from 80 to 300 nucleotides in length. F, forward primer; R, reverse primer.

Primer	Sequence
CCA1-F	CATTTCCGTAGCTTCTGGTCTCTT
CCA1-R	ATCAGCTTGGATTCGATAAAGATTC
LHY-F	TCCTCCATGGCTACTCTCAAGG
LHY-R	TCAGCAGCCAAACAGAGATCTTAG
LUX-F	AGCTTCGAAGAGCTCAATCTCTAACTGAA
LUX-R	TCGTAATCGCTCATTTGTACTTCCTCTC
TOC1-F	TGTTAAGGGGATAAATTAGGCGAC
TOC1-R	GCTATGATACTTCCATGGCCAAA
PRR7-F	TGGCCCGAGACAAATCTTTCTAATATCT
PRR7-R	GAGTGGAAATCGGAGACGACCATAA
PRR9-F	TCCAATTTGAATGATACATAGAGCAGCTG
PRR9-R	TGGGTTTCTATTGTAATTGTGTGGCTAAGT
MYB96(A)-F	CGACGTGCTTCGCAAATC
MYB96(A)-R	CAAACAAATACTCCAAACTGTAAAAC
MYB96(B)-F	GTTATCTCCACAAAATTAGAAACACG
MYB96(B)-R	CGACTTACACTCTAGTCTACAGTAATACG
MYB96(C)-F	GAGGAAGTTCACTAATTACGTAAAACTC
MYB96(C)-R	CGTGAATACATCCATTGAATTGTC
MYB96(D)-F	GTGTTATATTTAGTTTAGATCTTCACGCC
MYB96(D)-R	CAGGATGTGTGAGAGAAATAAAGG
MYB96(E)-F	GGAATTTTGAGTGTGTGAATTGATAG
MYB96(E)-R	CTCTTTCTCTCCAAAGTCAGCC
CCA1(A)-F	CTTCTCTTTGTATCACTTGAACCAA
CCA1(A)-R	GAATTTGAGTCTTCCATTCTCAGTATTA
CCA1(B)-F	GTCCTTCCTTCAATCTTTCCTTC
CCA1(B)-R	GCGCTATTCTCACACTCTAATGG
CCA1(C)-F	CAAGTTGATGTTAAGATGGACAAGAAT
CCA1(C)-R	CCACAAAACAAAAAACAATCAAGTC
LHY(A)-F	TAATTTAATTATATTGTTGTTAATGTATGTTTCA
LHY(A)-R	TTTGCTTAAAGCAGAAGCGTTTA
LHY(B)-F	GACCAAAGTTCTCGATGATAATTTG
LHY(B)-R	GCTTCAATGTGAAGCTCACGTAG
LHY(C)-F	TGTTTTGCTTCCGATTTGATTAT
LHY(C)-R	CTTCTCCAGCAGAACTCAAAAGA
LUX(A)-F	AATTTAATTATATTGTTGTTAATGTATGTTTCA
LUX(A)-R	TTTGCTTAAAGCAGAAGCGTTT
LUX(B)-F	TGACCAAAGTTCTCGATGATAATTT
LUX(B)-R	CAATGTGAAGCTCACGTAGGTAAA
LUX(C)-F	GATTATTTCCGGGAACGATGA
LUX(C)-R	CTTCTCCAGCAGAACTCAAAAGA
TOC1(A)-F	CTGATAAGAGTATCTGCATAGCTGTG
TOC1(A)-R	GTTTACGTATCTAACGAGTACTGCAAA
TOC1(B)-F	GGATAAAACCGATAATCGTGAGATAC
TOC1(B)-R	CTAAACTTTTATATAGAAAGTAAGTAGTCGACA
TOC1(C)-F	CCTAAAATTTGGTTTCTGATGGTT
TOC1(C)-R	CGGTGGAGATTAAGTCTTTTTCTTAC
PRR7(A)-F	GAATGTTCCTGAGGCAGAGTTG
PRR7(A)-R	GCGGTGCCTCCTCCTCT
PRR7(B)-F	CCTTTAGGTCAACGTATCTCGTG
PRR7(B)-R	GGTAAGGAAAACGTCAAGTGGT
PRR7(C)-F	GAATTCCTTGTTTCTATTTCCCAA
PRR7(C)-R	ATGATCAAAATATCAAAACACAGCC
PRR9(A)-F	TGAATCATGCTTGATTCTTTGTTT
PRR9(A)-R	CAACAAAAAAAAAGAAAGAATGTGAT
PRR9(B)-F	TTCGTTTATTATGATTATAAAATGTTTGATC
PRR9(B)-R	TTAAGCCTTAGATTTTCAAAAGCC
PRR9(C)-F	CGGCCACTAACGAAATTTGA
PRR9(C)-R	GCAGGTCCACCTTAACACGT

Supplementary Table S2. Primers used in ChIP assays.

F, forward primer; R, reverse primer.

Supplementary References

1. Voß, U. *et al*. The circadian clock rephases during lateral root organ initiation in *Arabidopsis thaliana*. *Nat. Commun.* **6**, 7641 (2015).
2. Seo, P. J. *et al*. The MYB96 transcription factor mediates abscisic acid signaling during drought stress response in *Arabidopsis*. *Plant Physiol*. **151**, 275-289 (2009).
3. Seo, P. J. *et al*. The MYB96 transcription factor regulates cuticular wax biosynthesis under drought conditions in *Arabidopsis*. *Plant Cell* **23**, 1138-1152 (2011).
4. Lee, H. G. & Seo, P. J. The MYB96-HHP module integrates cold and abscisic acid signaling to activate the CBF-COR pathway in *Arabidopsis*. *Plant J*. **82**, 962-977 (2015).
